# Micro-Injection Molding of Carbon-Fiber-Reinforced Plastic (CFRP)/Polymethyl Methacrylate (PMMA) Composite Components

**DOI:** 10.3390/polym16233338

**Published:** 2024-11-28

**Authors:** Yingying Xiao, Bin Xu, Hang Zhao, Likuan Zhu, Jianguo Lei

**Affiliations:** 1 Guangdong Provincial Key Laboratory of Micro/Nano Optomechatronics Engineering, Shenzhen University, 3688 Nanhai Avenue, Nanshan District, Shenzhen 518060, China; yingyxiao@foxmail.com (Y.X.); binxu@szu.edu.cn (B.X.); zhulikuan@szu.edu.cn (L.Z.); leijg@szu.edu.cn (J.L.); 2 Shenzhen Key Laboratory of High Performance Nontraditional Manufacturing, Shenzhen University, 3688 Nanhai Avenue, Nanshan District, Shenzhen 518060, China

**Keywords:** composite components, injection molding, carbon-fiber-reinforced plastic, polymethyl methacrylate, composite bonding

## Abstract

CFRP exhibits a low specific gravity, good rigidity, and high strength and is widely used in the automobile, aerospace, and biomedical fields. Against this background, the demand for composite components prepared using CFRP and polymers has increased. The service life of composite components is closely related to the bonding strength between the CFRP and the polymer. Here, using CFRP and polymethyl methacrylate (PMMA) as raw materials, composite components were prepared via injection molding. First, micro-grooves were produced on the CFRP surface using the hot-pressing technique. Subsequently, the melted PMMA was filled in these micro-grooves using injection molding, thereby forming the bonding interface of the composite components. These micro-grooves can increase the contact area between CFRP and PMMA, thereby enhancing the bonding strength of the CRFP and PMMA interface. In this study, a single-factor experiment was used to explore the influence of each process parameter on the tensile strength of the composite components. Finally, after optimizing process parameters, the composite components with tensile strength of 10.72 MPa were obtained.

## 1. Introduction

CFRP is a structural material produced via a bonding process with resin and carbon fiber. CFRP possesses the following properties: low density, high strength, good corrosion resistance, and good creep resistance. Moreover, it is one of the most advanced high-performance composite materials. Currently, CFRP is generally applied in the automobile and biomedical fields [1,2]. In practice, CFRP should be bonded with other materials to form composite components. Among the various materials connected to CFRP, polymers are widely used. Polymethyl methacrylate (PMMA) has been extensively used in construction, optical equipment, prosthodontics, and medical instruments due to its light weight, high transmittance, and good weather resistance [3,4]. PMMA is also a good choice for lightweight structure design and lightweight material use [5,6,7]. In this study, CFRP and PMMA were selected to prepare composite components because of their excellent properties. These components have a wide range of applications in automotive interior design. Furthermore, the research content of this study originated from the technical requirements of Shenzhen Silver Basis Technology Co., Ltd. (Shenzhen, China).

By combining the co-curing techniques with injection overmolding techniques, Miao et al. [8] fabricated the thermoset–thermoplastic structure. Li et al. [9] added nano-silica particles via the adhesive method, and the shear strength was enhanced by 88.292%. Using atmospheric pressure equipment, Wen et al. [10] processed the surface of the CF/EP, and its tensile shear strength was increased by 119.59% at most. During the riveting process, Altmeyer et al. [11] found that a 70% increase in the rivet tip width could produce a tensile force of 10.7 KN. To ensure the bonding strength, Ciftci [12] produced new CFRP materials that are lightweight and exhibit higher strength. Chen et al. [13] found that during the hybrid adhesive–rivet bonding of CFRP with aluminum alloys, a longer overlap length resulted in a higher peak load. By comparing three types of bonding technology, Jin et al. [14] found that hybrid bonding exhibited the best bonding quality. Using the brazing method, Hu et al. [15] used a Ti-Zr-Ni-Cu filler to vacuum braze the CRFP to titanium alloy. Using PA6 and 30% FG as raw materials, Nguyen et al. [16] fabricated polyamide fiberglass composites. Using laser joining, Jung et al. [17] were able to join CFRP and aluminum alloy via a high-quality bond. Applying ultrasonic vibration to the bonding of CFRP and aluminum alloy, Wu et al. [18] found that ultrasound techniques could form more chemical bonds, thereby promoting the bonding of aluminum alloys and adhesives. Liu et al. [19] joined PMMA and alumina ceramic through laser transmission welding, and the maximum joint strength was about 25 MPa. Huang et al. [20] welded PMMA and 304 austenitic stainless steel through laser welding experiments, and they obtained the optimal shear stress of 4.17 MPa.

**Figure 1:**
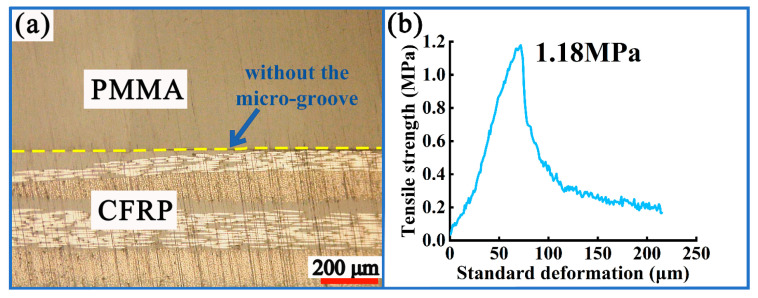
Composite components: (**a**) cross-section profile; (**b**) tensile curve.

Based on the above evidence, for composite components prepared from CFRP, the bonding strength between CFRP and other materials is a focus of the industry. Figure 1a shows a cross-section of a CFRP/PMMA composite component that was prepared using injection molding, and its tensile strength reached 1.18 MPa (Figure 1b). The tensile strength of CFRP/PMMA composite components currently does not meet application requirements in the automotive field. To further improve the tensile strength, micro-grooves were prepared on the CFRP surface in this study, and injection molding was used for the CFRP and PMMA, thereby obtaining composite components. Compared with the composite component displayed in Figure 1a, the micro-groove array was conducive to increasing the contact area between CFRP and PMMA, thereby improving its bonding strength. Therefore, the novelty of this paper is to prepare microstructures on the surface of CFRP and apply them to the preparation of CFRP/PMMA composite components in order to improve their tensile strength. The study employs a single-factor experimental method for the experimental design. The aim was to investigate the individual impact patterns of four process parameters that are closely related to molding quality. In this experimental scheme, other process parameters are kept constant to ensure that only the single experimental parameter of interest varies. By employing this method, the impact pattern and mechanism of the process parameter on tensile strength can be accurately analyzed, thereby optimizing process parameters. The results of this study can further improve the tensile strength of CFRP/PMMA composite components, which has important application value in the preparation of interior automotive composite components.

## 2. Materials and Methods

### 2.1. Materials and Experimental Equipment

As shown in Table 1, PMMA has a certain water-absorbing capacity. To avoid defects caused by the water-absorbing capacity, such as flow marks and voids, on the surface of the composite components, an appropriate drying process must be performed on PMMA. In this study, PMMA was dried in a drying oven to remove moisture. The drying temperature was 80 °C, and the drying duration was 4 h.

By using a slow wire-cutting machine (AP250L, Sodick, Yokohama, Japan), micro-grooves were machined on the surface of a hot-pressing mold. Via the hot-pressing technology, the CFRP surface can replicate micro-groove structures. Composite components comprising CFRP and PMMA were prepared. The bonding interface of the composite components was observed via a laser scanning confocal microscope (VK250, KEYENCE, Osaka, Japan). The tensile test on composite components was conducted using a universal testing machine (Z050, ZwickRoell, Ulm, Germany), and the tensile strength was obtained. An ultra-depth-of-field microscope (VHX-2000, KEYENCE, Japan) was used to test the fracture morphology of components.

### 2.2. Fabrication Process

**Figure 2:**
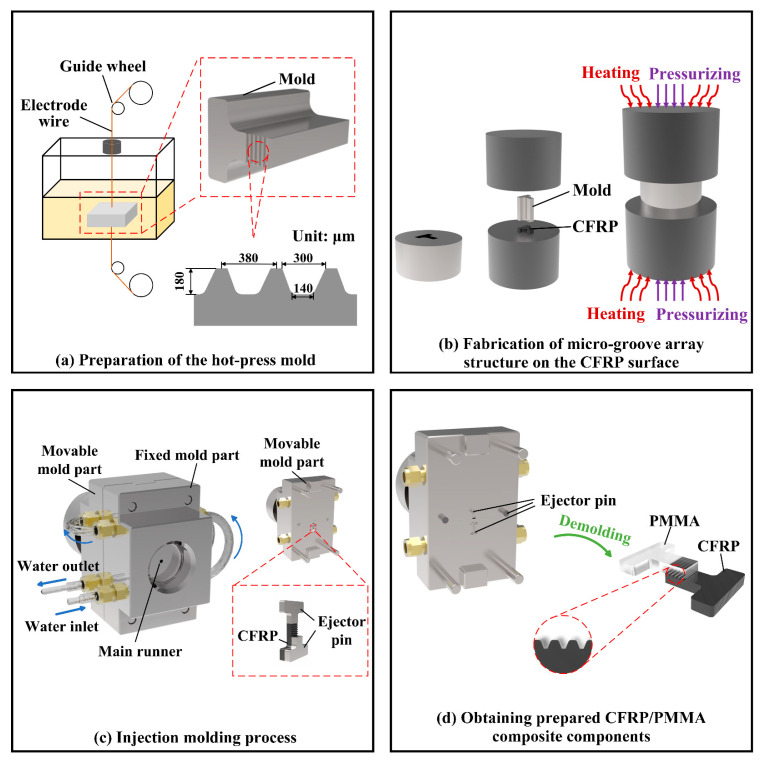
Preparation of the composite components.

The fabrication process adopted in this paper is described in Figure 2.

(1) A slow wire-cutting machine was used to prepare the mold. The micro-groove array structure was fabricated on the surface of the mold using a wire-cutting technique (Figure 2a).
(2) The mold and CFRP were placed in a hot press machine, and compression molding was carried out under a certain temperature, pressure, and time, thus completing the preparation of the micro-groove structure (Figure 2b).
(3) A gasket and the CFRP obtained from the above process were sequentially installed in the injecting mold. The dried PMMA was poured into the inlet of the injection molding machine. Then, through injection molding, the melted PMMA was filled into the micro-groove (Figure 2c) such that a tight connection was formed between the CFRP and PMMA (Figure 2d).

### 2.3. Design of Experiments

The injection molding process parameters include the melt temperature, mold temperature, injection speed, and holding pressure. These parameters are closely related to the molding quality. The purpose of this study was to analyze the individual effects of each process parameter on the forming quality of composite parts. In this manner, we aimed to examine the influence patterns and mechanisms of these four process parameters with respect to tensile strength, thereby obtaining more optimized process parameters. On this basis, a single-factor experimental method was used in this study (Table 2). The design of this experimental method is as follows: Within the same set of experiments, all process parameters remain constant except for a single process parameter under investigation. For the single process parameter being studied, different treatment levels are designed (such as varying temperatures). Subsequently, the experiment was carried out according to the designed scheme, and the relevant data of the experimental process were recorded. The experimental results of the cross-section profile, fracture morphology, and tensile curve were obtained. The influence of the process parameters on the tensile strength was obtained by analyzing the experimental results. The single-factor experiment method has the following advantages: It simplifies the problem and enhances experimental controllability. Firstly, by focusing on only one process parameter at a time, the specific effect of the process parameter on the experimental results can be clearly understood. Secondly, this method allows for better control of experimental conditions, as it ensures that only a single studied parameter changes while all other parameters remain constant. This improves the repeatability of the experiment and the reliability of the results. Therefore, the single-factor experiment method can achieve the experimental purpose of this study.

## 3. Results and Discussion

### 3.1. Melt Temperature

PMMA is a type of amorphous thermoplastic material. It is sensitive to temperature and pressure, softens when heated, and flows when subjected to external forces [21]. With increasing temperatures, PMMA exhibits three states sequentially: glassy state, rubbery state, and viscous state. During the injection molding process, the melted PMMA exhibits viscoelastic behaviors. This type of viscoelastic behavior mainly originates from the random coil configuration of melted polymer molecules, and this configuration allows molecular chains to slide under the impact of external loads. Viscosity is an important measure for describing the flow behavior of plastic melts, and it is impacted by various factors. The functional relationship describing polymer melt viscosity is shown in Equation (1):

η = F(γ,T,P,M,ΛΛ)(1)

where γ refers to the shear rate, T refers to the temperature, P refers to the static pressure, M refers to the molecular parameter, and ΛΛ refers to other influencing parameters.

As observed in Equation (1), temperature is an important factor affecting the melt viscosity. This is because as the temperature increases, the thermal motion of the polymer molecule increases. Under this condition, the interacting force between polymer molecules weakens, thus resulting in a decrease in the viscosity and an increase in the fluidity of the melt. In addition, activation energy refers to the energy needed per mole for a molecular chain to overcome intermolecular forces and change position during flow. The sensitivity degree of viscosity relative to temperature is proportional to activation energy. Because PMMA has relatively high activation energy, from this perspective, an increase in temperature can enhance the fluidity of PMMA.

To explore the proper melt temperature, we used different melt temperatures to perform injection molding experiments on CFRP specimens and PMMA. The melt temperatures were 240 °C, 245 °C, 250 °C, 255 °C, and 260 °C. Moreover, the mold temperature, injection speed, and holding pressure were set as 70 °C, 70 mm/s, and 8 MPa, respectively.

**Figure 3:**
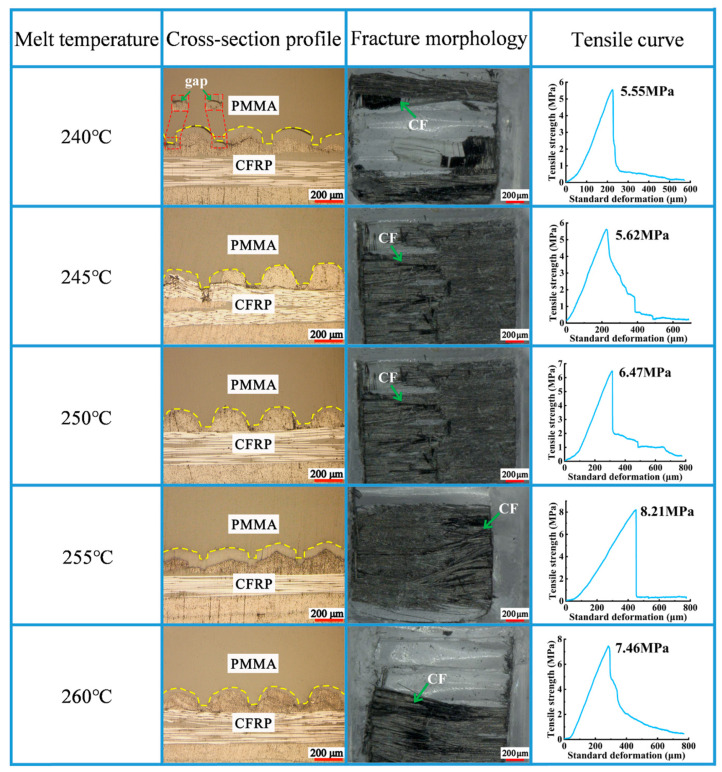
Impact of melt temperature on molding quality of the composite components.

**Figure 4:**
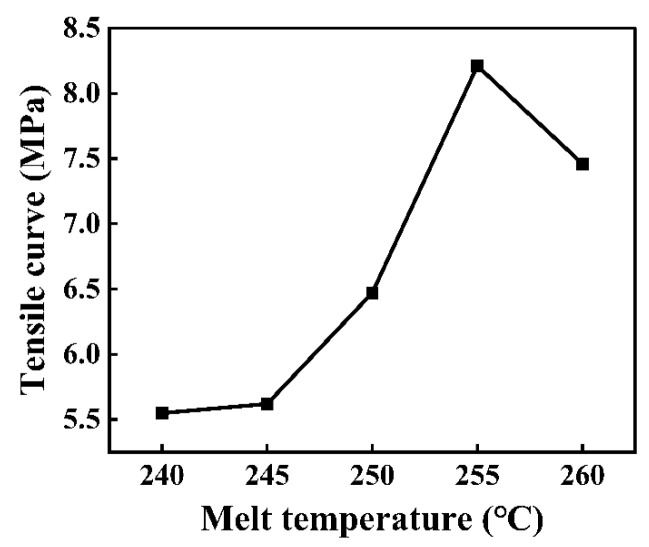
Impact of melt temperature on tensile curve of the composite components.

As shown in Figure 3, PMMA could be filled in the micro-groove, and the molding quality was good. The tensile strength reached 5.55 MPa with a melt temperature of 240 °C. At this point, the melt temperature was low, and the melted PMMA exhibited high and low viscosities; thus, PMMA could not be effectively filled into the micro-groove. Under these conditions, a good bonding interface could not be formed between PMMA and CFRP, and the tensile strength was unsatisfactory. Within the range of 240 °C to 255 °C, the tensile strength increased with an increase in melt temperature, which is shown in Figure 4. With increasing melt temperatures, the free volume of PMMA molecules increases; consequently, the distance between molecules increases, resulting in a decrease in molecular interactions. Furthermore, the melt viscosity is very sensitive to temperature change. An increase in temperature can considerably decrease the melt viscosity and result in good fluidity, and PMMA can be more effectively filled into the micro-groove. Under these conditions, a good bonding interface could be formed between PMMA and CFRP; therefore, the tensile strength was enhanced. When the melt temperature further reached 260 °C, the tensile strength decreased to 7.46 MPa. An excessively high melt temperature can increase the cooling time to some extent, thereby resulting in the uneven shrinkage of PMMA. Under the impacts of the abovementioned factors, the tensile strength could decline to some extent.

### 3.2. Mold Temperature

During the fabrication of the composite components, the PMMA entering the high-temperature barrel was heated and melted. Under the action of pressure, the melted PMMA was injected into the mold and filled in the micro-groove. If the temperature of the mold was not controlled, the melted PMMA would contact the mold while entering the mold cavity, producing convective heat transfer. At this moment, the heat dissipation of the melted PMMA resulted in a decrease in temperature, causing an increase in the flow resistance of the melted PMMA. Under the impact of the abovementioned factors, the flow rate of the melted PMMA was reduced, which hindered continuous material feeding and finally resulted in the appearance of short shots on the composite components.

To obtain proper mold temperatures, we performed injection molding experiments under different mold temperatures. The mold temperatures were set to 60 °C, 65 °C, 70 °C, 75 °C, and 80 °C. During the experiment, the melting temperature, injection speed, and holding pressure were set to 255 °C, 70 mm/s, and 8 MPa, respectively.

**Figure 5:**
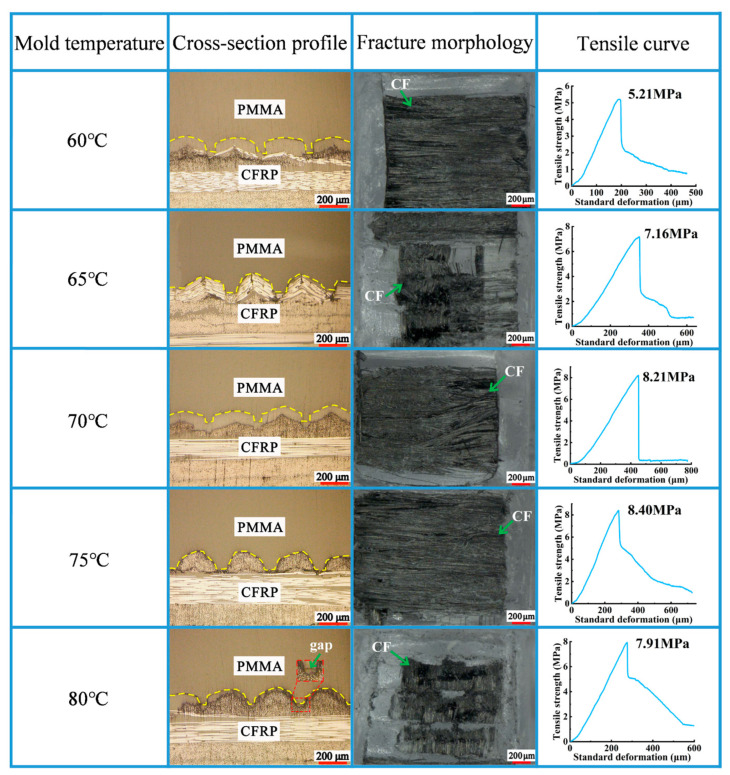
Impact of mold temperature on molding quality of the composite components.

As observed in Figure 5, with the gradual increase in mold temperature from 60 °C to 75 °C, the tensile strength gradually increased to 8.4 MPa. With respect to the results in Section 3.1, an increase in the melt temperature could reduce the viscosity of the PMMA, thereby improving the fluidity of the melt. An increase in the mold temperature can provide substantial heat for the mold wall, partially compensating for the heat loss produced via convective heat transfer. Therefore, an increase in the mold temperature could slow the temperature decline rate of the melt in the mold cavity. Furthermore, an increase in mold temperatures could effectively reduce the thickness of the solidified material layer generated on the mold wall during the filling process, thereby reducing the filling resistance of the melt.

**Figure 6:**
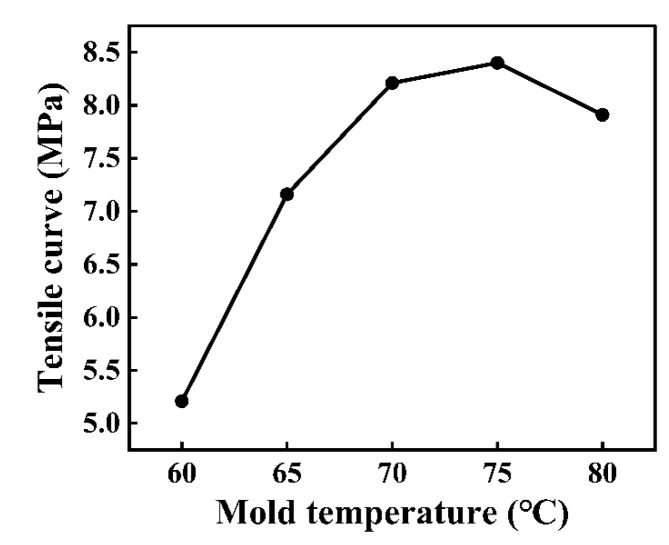
Impact of mold temperature on tensile curve of the composite components.

High temperatures are conducive to cell stacking, reducing the defects inside the cells and finally obtaining composite components with low internal stress. With the mold temperature further increasing from 75 °C to 80 °C, the tensile strength decreased to 7.91 MPa (Figure 6). At 80 °C mold temperature, gaps appeared at the bonding interface of CFRP and PMMA. When the mold temperature was excessively high, the cooling time increased. Consequently, the molding’s shrinkage rate increased, resulting in a large sample deformation after demolding. Consequently, the melted PMMA could not be filled in the micro-groove, thus generating gaps at the bonding interface. Based on the above experimental results, the mold temperature was set to 75 °C.

### 3.3. Injection Speed

The injection speed refers to the moving speed of the feed screw or the hydraulic cylinder piston rod of the injection molding machine. During the injection molding process, the speed of the melt entering the mold cavity has an impact on the properties of the composite components. To obtain the appropriate injection speed, experiments were conducted under different injection speeds. The holding pressure, mold temperature, and melt temperature were 8 MPa, 75 °C, and 225 °C.

**Figure 7:**
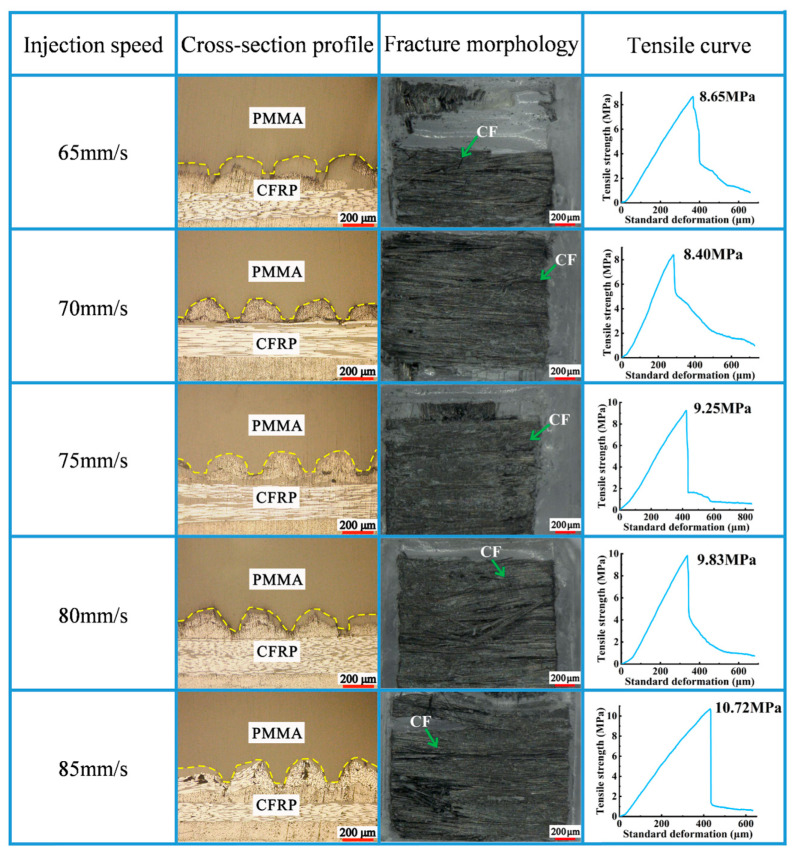
Impact of injection speed on molding quality of the composite components.

**Figure 8:**
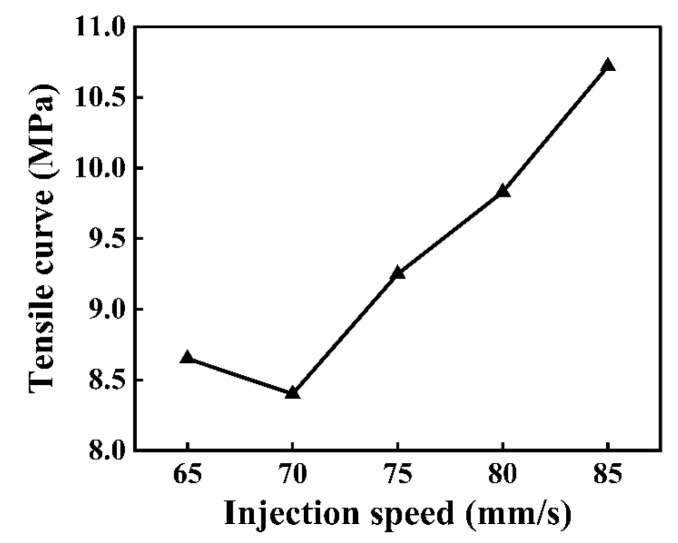
Impact of injection speed on tensile curve of the composite components.

As shown in Figure 7, the tensile strength showed an overall increasing trend with an increase in the injection speed. The largest tensile strength (10.72 MPa) of the composite components was obtained under an injection speed of 85 mm/s (Figure 8). The injection pressure is proportional to the injection speed. From Figure 7, when the injection speed was relatively high, high injection pressures could be achieved on the melted PMMA. Under this condition, the melted PMMA could be sufficiently filled in the micro-groove array structure on the CFRP surface, thereby obtaining high-quality composite components.

When the injection speed was low, the time of contact between the melt and mold cavity wall was long. At this moment, the heat loss of the melt through the mold cavity wall increased, resulting in a decrease in melt fluidity. Under the impact of the abovementioned factors, the melt PMMA could not be sufficiently filled in the micro-groove. When injection speeds increased continuously within an appropriate range, the shear rate of the melt increased, which was conducive to shear heating and resulted in an increase in melt temperatures. Under the impact of the abovementioned factors, a decline in the viscosity of the melt was favorable for the enhancement of molding quality.

### 3.4. Holding Pressure

The pressure applied to compensate for the volume reduction in the mold cavity caused by the cooling and shrinkage of PMMA is called holding pressure. The experiments were performed under different holding pressures to explore the proper holding pressure (as shown in Figure 6). The injection speed, mold temperature, and melt temperature were set as 85 mm/s, 75 °C, and 225 °C.

**Figure 9:**
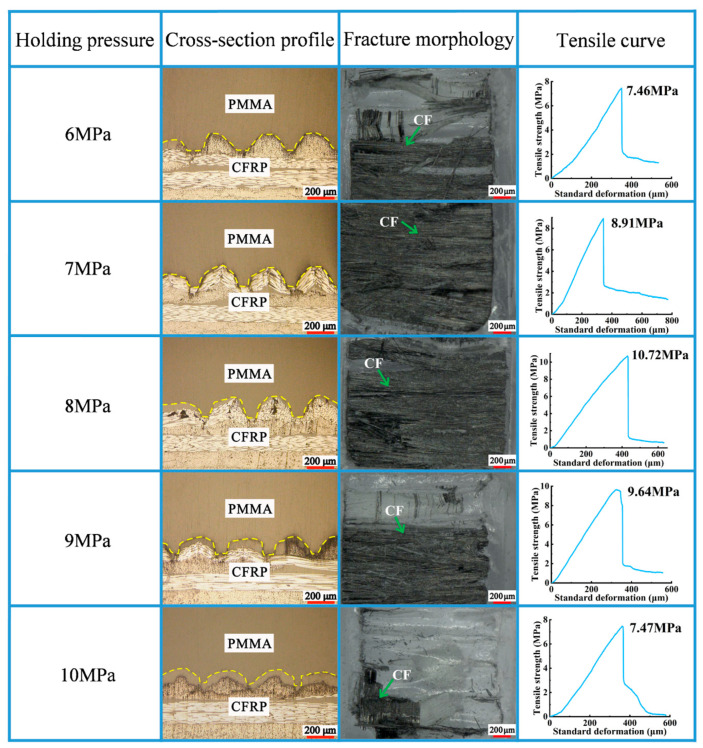
Impact of holding pressure on molding quality of the composite components.

**Figure 10:**
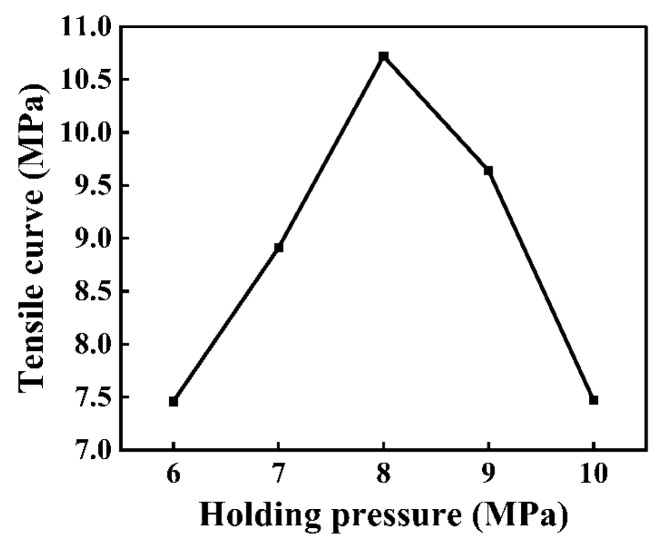
Impact of holding pressure on tensile curve of the composite components.

With a gradual increase in holding pressure from 6 MPa to 8 MPa, the tensile strength increased from 7.46 MPa to 10.72 MPa (Figure 9). As shown in Figure 10, when the tensile strength was 10.72 MPa, the rupture cross-section contained more carbon fibers. With the holding pressure continuously reaching 10 MPa, the tensile strength dropped to 7.47 MPa. When holding pressures were excessively low, shrinkage due to the cooling of the melted PMMA resulted in a poor bond between the PMMA and the micro-groove. Appropriate holding pressures can compensate for the volume shrinkage produced during the cooling of the melted PMMA. Additionally, an appropriate holding pressure can ensure that the melted PMMA can sufficiently enter the mold cavity such that a good bonding interface is formed. Excessively high holding pressures can result in overly high internal stress on the composite components, resulting in the appearance of defects, such as flashes and deformations.

## 4. Conclusions

To enhance the molding quality of the bonding interface of CFRP/PMMA composite components, we used CFRP with a micro-groove on its surface and bonded it with PMMA via injection molding, thereby obtaining composite components with high tensile strength. The main conclusions are as follows:

(1) Using hot pressing technology and micro-injection molding technology, CFRP/PMMA composite components were successfully fabricated. The experimental results show that this method can effectively improve the tensile strength of composite components (from 1.18 MPa to 10.72 MPa). These results are mainly attributed to the preparation of micro-grooves on the surface of CFRP. These micro-grooves can increase the contact area between CFRP and PMMA, thereby enhancing the bonding strength of the CRFP and PMMA interface.
(2) Based on an analysis of the single-factor experiment, it was observed that the injection molding process parameters had a distinct influence on the tensile strength of PMMA in this experiment. Specifically, as the melt temperature, mold temperature, and holding time increased, the tensile strength of PMMA exhibited an initially increasing trend that then decreased, reaching the peak tensile strength value under a melt temperature of 260 °C, a mold temperature of 75 °C, and a holding pressure of 8 MPa. Conversely, the tensile strength of PMMA exhibited an initially decreasing trend that increased with respect to an increase in injection speed, and it reached the maximum value at an injection speed of 85 mm/s.
(3) According to the experimental results, with a melt temperature, mold temperature, injection speed, and holding pressure of 255 °C, 75 °C, 85 mm/s, and 8 MPa, relatively optimized process parameters can be obtained. Further optimizations and mechanism analyses of this process will be carried out in future studies.

## Figures and Tables

**Table 1:** Physical properties of PMMA.

Density (g/cm^3^)	Tensile Strength (MPa)	Water Absorbing Capacity (%)
1.19	70	0.3

**Table 2:** Parameters of injection molding experiments on the composite components.

Sequence Number	Melt Temperature (°C)	Mold Temperature (°C)	Injection (mm/s)	Hold Pressure (MPa)
1	240	70	70	8
2	245	70	70	8
3	250	70	70	8
4	255	70	70	8
5	260	70	70	8
6	255	60	70	8
7	255	65	70	8
8	255	75	70	8
9	255	80	70	8
10	255	75	65	8
11	255	75	75	8
12	255	75	80	8
13	255	75	85	8
14	255	75	85	6
15	255	75	85	7
16	255	75	85	9
17	255	75	85	10

## Data Availability

The original contributions presented in the study are included in the article, further inquiries can be directed to the corresponding author.
